# 2022 Peritoneal Surface Oncology Group International Consensus on HIPEC Regimens for Peritoneal Malignancies: Colorectal Cancer

**DOI:** 10.1245/s10434-023-14368-5

**Published:** 2023-11-08

**Authors:** Martin Hübner, Kurt van Der Speeten, Kim Govaerts, Ignace de Hingh, Laurent Villeneuve, Shigeki Kusamura, Olivier Glehen

**Affiliations:** 1 Department of Visceral Surgery, Lausanne University Hospital (CHUV), University of Lausanne (UNIL), Lausanne, Switzerland; 2 Department of Abdominal and Oncological Surgery, Ziekenhuis Oost Limburg (ZOL), Genk, Belgium; 3 Department of Epidemiology, GROW School for Oncology and Developmental Biology, Maastricht University, Maastricht, The Netherlands; 4 Department of Surgery, Catharina Cancer Institute, Eindhoven, The Netherlands; 5 Fondazione IRCCS Istituto Nazionale dei Tumori, Milan, Italy; 6 Department of Surgical Oncology, Centre Hospitalier Lyon-sud, Lyon, France; 7 CICLY: Center for Innovation in Cancer in Lyon, University Lyon 1, Lyon, France

**Keywords:** Peritoneal metastases, Peritoneal surface malignancies, Cytoreductive surgery, Hyperthermic intraperitoneal chemotherapy, Treatment regimens

## Abstract

**Background:**

Selected patients with peritoneal metastases of colorectal cancer (PM-CRC) can benefit from potentially curative cytoreductive surgery (CRS) ± hyperthermic intraperitoneal chemotherapy (HIPEC), with a median overall survival (OS) of more than 40 months.

**Objective:**

The aims of this evidence-based consensus were to define the indications for HIPEC, to select the preferred HIPEC regimens, and to define research priorities regarding the use of HIPEC for PM-CRC.

**Methods:**

The consensus steering committee elaborated and formulated pertinent clinical questions according to the PICO (patient, intervention, comparator, outcome) method and assessed the evidence according to the Grading of Recommendation, Assessment, Development, and Evaluation (GRADE) framework. Standardized evidence tables were presented to an international expert panel to reach a consensus (4-point, weak and strong positive/negative) on HIPEC regimens and research priorities through a two-round Delphi process. The consensus was defined as ≥ 50% agreement for the 4-point consensus grading or ≥ 70% for either of the two combinations.

**Results:**

Evidence was weak or very weak for 9/10 clinical questions. In total, 70/90 eligible panelists replied to both Delphi rounds (78%), with a consensus for 10/10 questions on HIPEC regimens. There was strong negative consensus concerning the short duration, high-dose oxaliplatin (OX) protocol (55.7%), and a weak positive vote (53.8–64.3%) in favor of mitomycin-C (MMC)-based HIPEC (preferred choice: Dutch protocol: 35 mg/m^2^, 90 min, three fractions), both for primary cytoreduction and recurrence. Determining the role of HIPEC after CRS was considered the most important research question, regarded as essential by 85.7% of the panelists. Furthermore, over 90% of experts suggest performing HIPEC after primary and secondary CRS for recurrence > 1 year after the index surgery.

**Conclusions:**

Based on the available evidence, despite the negative results of PRODIGE 7, HIPEC could be conditionally recommended to patients with PM-CRC after CRS. While more preclinical and clinical data are eagerly awaited to harmonize the procedure further, the MMC-based Dutch protocol remains the preferred regimen after primary and secondary CRS.

**Supplementary Information:**

The online version contains supplementary material available at 10.1245/s10434-023-14368-5.

Peritoneal metastases (PM) are diagnosed in 4–15% of patients with colorectal cancer (CRC) at the time of diagnosis and in up to 25% of patients presenting with disease recurrence.^1^ These patients’ prognoses are dismal and overall survival (OS) is worse for PM than for other metastatic sites.^1–3^ Of note, first-line palliative chemotherapy improvements alone have been modest over time, remaining below expectations from other settings, reaching 16.3 months. Taking into account lead-time bias and improvements in locoregional treatments and palliative care, no significant advantage has been made for palliative chemotherapy, the second-line of treatment, except for 5% of patients eligible for immunotherapy.^2,4–6^ The limited response of PM to systemic chemotherapy has been explained by pharmacokinetic limitations and, more recently, by a specific molecular profile suggesting a distinct disease entity.^1,6,7^ Cytoreductive surgery (CRS) offers a median OS beyond 40 months, with a potential for cure in selected patients.^6,8,9^ Patient selection, considering the biological profile and mutational status, as well as surgical and perioperative care performed in expert centers, are all required to achieve these optimal outcomes.^6,8,10,11^ Supported by a strong pharmacological rationale, hyperthermic intraperitoneal chemotherapy (HIPEC) has been proposed as an adjunct treatment after complete CRS, taking advantage of improved pharmacokinetics in order to address residual microscopic disease residuals.^1,6^ However, three recent randomized trials failed to demonstrate survival benefits by adding short-duration, high-dose oxaliplatin HIPEC in the adjuvant and prophylactic settings.^9,12,13^ Despite limited evidence, most expert centers continued to offer HIPEC but switched to alternative regimens, mainly including mitomycin-C (MMC).^14–17^ Currently, over 60 protocols differing in drug, concentration, duration, fractioning, and carrier solution are available, rendering comparisons between experiences very challenging.^18^ This triggered the Peritoneal Surface Oncology Group International (PSOGI) to launch an evidence-based consensus process to harmonize HIPEC protocols and define the most interesting questions for future research.^19^

## Methods

The consensus process on HIPEC regimens for PM of CRC (PM-CRC) followed the methodology that has recently been published.^20^ The essential components were as follows.

### Formation of the Guideline Development Group, Definition of the Timeline

The Steering Group of eight experts appointed the section leader and three additional experts to form the Colorectal Working Group (MH, KvS, KG, IdH). The general technical aspects of HIPEC were treated for all disease entities by a specific working group. Three core group members (LV, SK, OG) were involved to ensure consistency and to avoid overlap with the other chapters. A 12-month timeline was defined until the completion of the consensus statement.

### Definition of Scope and Formulation of Delphi Questions

The core group elaborated a consensus statement in three different sections:

(A) *Evidence-based recommendations* by use of the Grading of Recommendation, Assessment, Development, and Evaluation (GRADE) system and Patient, Intervention, Comparator, and Outcome (PICO) method.
(B) *Opinion survey* on the current practice with regard to indications for HIPEC and choice of the protocol in typical clinical scenarios.
(C) *Research recommendations* to identify priority topics and optimal methodology in the field of HIPEC for colorectal PM.

### Systematic Review of the Literature and Grading of Evidence

The working group conducted a Medline and Embase database search (search period 1 January 1985–1 February 2021) for randomized and non-randomized studies on HIPEC regimens using the following Medical Subject Heading (MeSH) search terms: ‘hyperthermic intraperitoneal chemotherapy’, ‘IPHP’, ‘IHCP’, ‘CHIP’, ‘HIPEC’, ‘colorectal cancer’, ‘carcinomatosis’, ‘peritoneal metastasis’, ‘randomized trial (RCT)’, ‘meta-analysis’, ‘prospective study’, and ‘comparative study’. The literature research was completed by manual review and cross-referencing. Eligible studies were assessed for quality and risk of bias, and the relevant data endpoints were entered into structured evidence tables to objectively present the available evidence to the expert panel.^20^

### Independent Expert Review

The core group cross-checked Delphi questions and evidence tables for content and consistency. Controversies were discussed and settled during two virtual meetings between the core group and the Colorectal Working Group.

### Two-Round Delphi Process

The core group invited medical and surgical oncologists, as well as visceral surgeons, being experts (>100 CRS±HIPEC procedures) in the management of peritoneal surface malignancies (PSMs), to serve as the expert panel. All geographic areas were considered and no center was voluntarily excluded.

Comprehensive text sections were provided with evidence tables and references for the different chapters through an online survey (SurveyMonkey Inc., San Mateo, CA, USA). Questions were provided as multiple-choice questions or voting on a 4-point Likert scale (strong positive [SP], weak positive [WP], weak negative [WN], strong negative [SN]). The results of the first Delphi round were provided to the expert panel for Delphi round 2. Experts had at least 3 weeks to complete each round and received reminders before the closure of consensus voting. Consensus was defined as > 50% agreement for the 4-point consensus grading, or as >70% for the combination (weak and strong positive or negative, respectively) or binary questions.

## Results

The consensus process was completed according to the predefined protocol and timeline. The working group considered the following content (29 questions in total) as essential for the consensus process:

(A) *Evidence-based recommendations:* Ten PICO questions on HIPEC regimens for colorectal PM.
(B) *Opinion survey:* Three questions on indications and three questions on the choice of HIPEC protocols to be used in clinical practice for colorectal PM.
(C) *Research recommendations:* Of 13 questions in total, five related to indications and five related to methodology of the respective study, two were on HIPEC regimens, and one was on risk factors for the development of colorectal PM.

In total, the systematic literature research revealed 60 articles retained for the text sections; they are comprehensively summarized in the respective Summary of Findings (SoF) Tables (Tables 1–13, Online Resource Appendix). Overall, evidence was low or very low for most predefined questions.

**Fig. 1:**
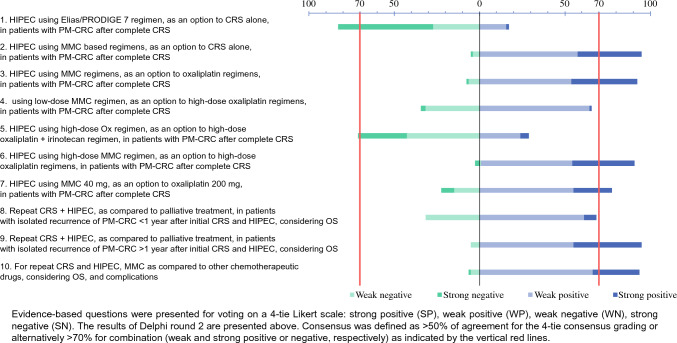
Expert consensus on HIPEC regimens for colorectal peritoneal metastases

Overall, 119/145 invited experts accepted the invitation and actively participated in the Delphi process. Ninety panelists were eligible for participation in the colorectal chapter, and 80 and 70 completed Delphi rounds 1 and 2, respectively, with a final response rate of 78% (Table 14, Online Resource Appendix).

(A) *Evidence-based recommendations* are summarized in Fig. 1. Consensus was reached for 10/10 questions, with weak consensus reached for eight questions and one each for strong and combined consensus. Notably, >70% of the combined (weak and strong) agreement was reached in 8/10 items. The following paragraphs provide the evidence that was presented to the experts for voting.

### Question 1: Role of Hyperthermic Intraperitoneal Chemotherapy (HIPEC) Using the High-Dose Oxaliplatin (Elias/PRODIGE 7) Regimen?

The PRODIGE 7 trial did not show survival benefits when adding OX-based HIPEC according to the Elias regimen (high dose, short duration) after complete CRS.^9^ Severe complications at 60 days postoperatively were higher (odds ratio [OR] 1.99) in the HIPEC arm. Only a subgroup of patients with an intermediate Peritoneal Cancer Index (PCI; 11–15) appeared to have a survival benefit with the addition of HIPEC. Of note, OS in both groups was unexpectedly high, underlining the important role of high-quality CRS and selection by systemic chemotherapy.

### Question 2: Role of HIPEC Using Mitomycin-C in Colorectal Cancer (CRC) [Online Resource Tables 1 and 2]

Three comparative observational studies analyzed the impact of MMC-based HIPEC in addition to CRS.^21–23^ Including a total of only 225 patients, those patients receiving additional HIPEC had a significantly better survival with no increased complication rate. For the sake of completeness, MMC was combined with cisplatin (CDDP) in one study,^23^ while in another study it was administered alone or in combination with CDDP,^21^ and in the last study, MMC was followed by 5-FU early postoperative intraperitoneal chemotherapy (EPIC).^22^ Interestingly, Baratti et al. also observed a high median OS of 39.3 months in the group receiving systemic treatment and CRS, which did not significantly differ from the HIPEC group.^21^ However, unlike in PRODIGE 7, administration of MMC-based HIPEC was not associated with a higher severe complication rate.

### Question 3: Mitomycin-C or Oxaliplatin as the HIPEC Regimen for Peritoneal Metastases of Colorectal Cancer (PM-CRC) [Online Resource Tables 3 and 4]

Eleven comparative observational studies compared MMC-based HIPEC regimens with OX-based HIPEC regimens. Dosage varied considerably for MMC (12.5–40 mg/m^2^) and OX (200–460 mg/m^2^), which was combined with irinotecan in one of the studies.^24–34^ Recently, Zhang et al. performed a systematic review and meta-analysis of comparative studies regarding these two HIPEC regimens and concluded there was no survival benefit for either regimen over the other. Significantly more complications occurred in patients receiving OX-based HIPEC.^16^

### Question 4: High-Dose Oxaliplatin Versus Low-Dose Mitomycin-C (Online Resource Tables 5 and 6)

In three observational studies, high-dose oxaliplatin (350–460 mg/m^2^) was compared with low-dose MMC (10–15 mg/m^2^), showing significantly better OS in favor of high-dose OX HIPEC, with no difference in morbidity.^31–33^ According to three observational comparative studies of low evidence, a survival advantage for high-dose OX compared with low-dose MMC, with no difference in morbidity, can be concluded.

### Question 5: High-Dose Oxaliplatin Versus High-Dose Oxaliplatin + Irinotecan (Online Resource Tables 7 and 8)

Quenet et al. studied the addition of irinotecan to high-dose OX in a multicenter observational study; patients receiving the combination treatment had more severe complications but no advantage in survival.^24^

### Question 6: High-Dose Oxaliplatin Versus High-Dose Mitomycin-C (Online Resource Tables 9 and 10)

In four observational studies, the high-dose OX regimen was compared with high-dose MMC, with no difference in survival but more severe complications in OX-treated patients.^26,29,30,34^

### Question 7: Oxaliplatin 200 mg 120 min Versus Mitomycin-C 40 mg (Online Resource Table 11)

Only one observational study compared low-dose OX HIPEC (200 mg, 120 min) with high-dose MMC (40 mg) showing no difference in survival but more severe complications in the OX-treated patients.^28^

### Questions 8, 9, and 10: Role of Repeat CRS/HIPEC (Online Resource Tables 12 and 13)

Only two studies reported the outcome of CRS and HIPEC after repeat CRS and HIPEC for isolated recurrence and compared this with palliative systemic treatment.^34,35^ Other studies only reported follow-up after second CRS and HIPEC and did not have a control group.^24,36,37^

(B) *Opinion Survey*

**Fig. 2:**
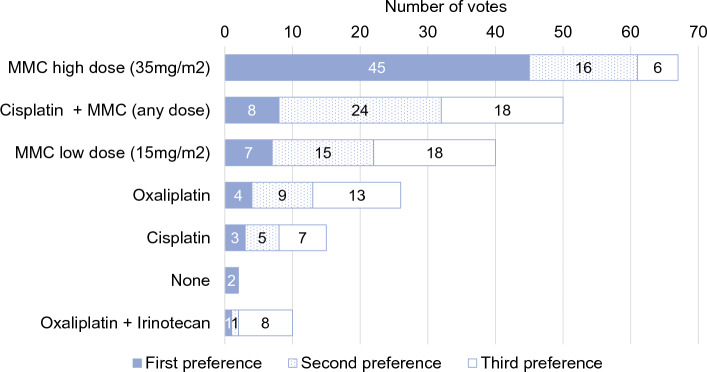
HIPEC regimen for patients with colorectal peritoneal metastases who were not treated with systemic therapy in the 6 months prior to CRS and HIPEC

**Fig. 3:**
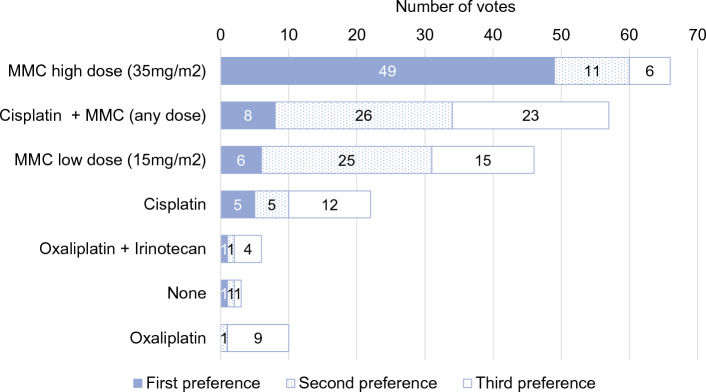
HIPEC regimen for patients with colorectal peritoneal metastases who were treated with oxaliplatin-containing systemic therapy (either in adjuvant or neo-adjuvant setting) in the 6 months prior to CRS and HIPEC

**Fig. 4:**
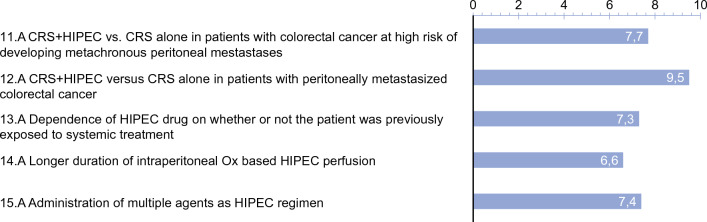
Research priorities to optimize HIPEC treatment

Seventy-three experts (91%) voted in favor of adding HIPEC after primary CRS. Seventy-seven panelists (96%) stated that late recurrence should be an indication for re-do CRS+HIPEC (≥1 year after index surgery), while 45 (64%) experts would not perform CRS+HIPEC for early recurrence (<1 year). The MMC-based Dutch protocol (35 mg/m^2^, 90 min, three fractions, 41°C) was the first choice in patients without prior chemotherapy (*n* = 45, 64.3%) (Fig. 2), in patients with prior OX-based systemic chemotherapy (*n* = 49, 70%) (Fig. 3), and for iterative CRS+HIPEC (*n* = 38, 54.3%). The second and third choices for these three indications were CDDP+MMC and low-dose MMC (15 mg/m^2^) [Fig. 5, Online Resource Appendix].

(C) *Research priorities* are summarized in Fig. 4. The panelists received a succinct overview on the existing evidence to facilitate their voting.

1. Prophylactic HIPEC

Risk factors associated with the development of PM have been described with variable attributed importance.^12,38–40^ Although a recent randomized controlled trial (RCT) on prophylactic HIPEC (the Elias regimen) could not demonstrate a 3-year OS or disease-free survival (DFS) difference, it did reveal an incidence of PM of 52% that was missed by standard modern imaging.^12^ COLOPEC compared the use of adjuvant HIPEC (the Elias regimen) combined with systemic chemotherapy with adjuvant systemic treatment alone after resection of a pT4 or perforated CRC. No difference in peritoneal-free survival could be appreciated at diagnostic laparoscopy 18 months later. In the control and intervention groups, 23% and 19% developed PM, respectively.^38^

Of note, the Spanish multicenter HIPEC T4 trial^21^ had not been presented or published at the time of the consensus.

2. Role and Impact of Systemic Chemotherapy, Indication and Regimen for Adjuvant HIPEC After Complete CRS

Almost all patients in PRODIGE 7 received systemic chemotherapy with or without targeted therapy. For the CRS/HIPEC group, the timing related to surgery was before surgery in 23% of patients, after surgery in 12% of patients, and pre- or postoperatively in 60% of patients. Therefore, most of the included patients received neoadjuvant OX-based systemic chemotherapy.^9^ Evidence for an impact on CRC cell biology after exposure to systemic OX is increasing, especially after administration within 2 months prior to HIPEC.^41–46^ Furthermore, Nagourney et al. also reported 5-FU resistance, while the activity for MMC or irinotecan was not significantly influenced by OX-based systemic treatment.^43^ Additional data regarding chemosensitivity/resistance after neoadjuvant OX (but not for MMC, 5-FU, or CDDP) have previously been presented as abstracts and are expected to be published in the near future. Lemoine et al. showed that after 30 min of hyperthermic OX perfusion (460 mg/m^2^ with 400 mg/m^2^ 5-FU + Leucovorin (LV)), the majority of the chemotherapeutic agent was removed with subsequent drainage of the peritoneal cavity (62.37 ± 24.41% for the body surface area-based regimen).^47^ Interestingly, Lemoine et al. also described an increase in plasma levels of between 90 and 330 min after the start (and thus cessation) of HIPEC, indicating a redistribution phase. Furthermore, in a recently published report on pharmacologic monitoring regarding upfront CRS in chemotherapy-naive patients undergoing intravenous and intraperitoneal perioperative chemotherapy administration, including the HIPEC component at a lower dose over a longer period of time; at the end of treatment, 85–90% of the OX had cleared from the peritoneal space.^41^ An earlier publication on the administration of MMC over a period of 90 min observed that the majority was also retained within the patient’s body.^48^

It is probably sensible to state that the response of CRC to a single chemotherapeutic agent approximates to 20%.^41,48–50^ Regarding systemic treatment, the utilization of multiple drugs resulted in a significant positive effect on both DFS and OS (MOSAIC RCT).^51,52^ For example, FOLFOX relates to the administration of 130 mg/m^2^ of OX (400 mg/m^2^ leucovorin) over 120 min, with a 2400 mg/m^2^ 5-FU infusion over 46 h. Knowing it is likely that around 20% of CRCs are refractory to OX, it is estimated that two-thirds of the treatment effect can be attributed to 5-FU.^49,50,53–55^ Most of the current HIPEC regimens only relate to a single agent intraperitoneally.

Interestingly, an RCT was voted to be the preferred study design for evaluation of (1) prophylactic HIPEC in CRC with high risk of developing PM (68, 97%); (2) choice of HIPEC regimen according to prior exposure to systemic therapy (58, 83%); (3) longer exposure of OX-based HIPEC; and (4) multiple-agent HIPEC regimens (61, 87%). The panel confirmed eight frequently reported risk factors for metachronous PM, with ≥70% of the votes for seven of eight of these risk factors (mucinous histology, 53%). The top three choices for HIPEC regimens to be investigated within the framework of clinical trials in the therapeutic and prophylactic setting are provided in Fig. 6 (online appendix). The study of CRS versus CRS+HIPEC deserves special mention. While this ongoing controversy was voted essential for future research by 60 experts (85%), all but 2 (97%) proposed comparative non-randomized studies to answer this question.

## Discussion

The last 20 years has seen the publication of two seemingly conflicting RCTs on the added value of HIPEC after CRS in patients with PM-CRC.^9,56,57^ Whereas mitomycin-based HIPEC from the Dutch trial^57,58^ demonstrated a significant survival benefit of CRS plus HIPEC versus CRS alone, the OX-based PRODIGE-7 trial^9^ was not able to reveal any benefit of HIPEC. Both trials were hampered by methodological and pharmacological flaws but established beyond doubt the survival benefit of complete and standardized CRS in PM-CRC patients. As the HIPEC regimen is currently not standardized, this needs further validation in both preclinical and clinical trials. This Delphi-based consensus aimed to identify the role of HIPEC and the best-suited HIPEC regimens for clinical practice as well as future investigation.^20,59^

As a direct result of the PRODIGE 7 trial, the expert panel (67.5%, SN, level of evidence [LoE] high) recommended (recommendation 1, R1) discontinuation of the high-dose, 30-min, OX-based HIPEC regimen in PM-CRC, which is in line with both historical and recent pharmacological research demonstrating this regimen to be ineffective.^47,60^ R2 (95.0%, WP, LoE low) and R3 (92.5%, WP, LoE low) both confirmed this post-PRODIGE 7 shift towards MMC-based HIPEC regimens as the preferred alternative for OX. However, the available low-quality evidence does not report strikingly better outcomes for MMC in terms of survival and morbidity.^17^ The systematic review and meta-analysis of 11 comparative MMC versus OX observational studies by Zhang et al. does not reveal a survival benefit in favor of MMC; MMC’s only advantage was the safety profile.^16^ Experts abandoned high-dose OX-based HIPEC compared with low-dose MMC, even though there was a survival advantage in favor of low-dose MMC (R4, 64.3%, WP, LoE low). Furthermore, adding irinotecan to the high-dose OX HIPEC regimen did not change the R5 panel recommendation (71.3%, WN+SN, LoE low) on discouraging high-dose OX-based HIPEC regimens. High-dose OX-based HIPEC is associated with higher morbidity, including increased bleeding risk.^61–63^ According to GRADE, the recommendations delivery process is a function of several factors, the most important of which is the balance between benefits and harms. There was an over-reliance on MMC-based regimens, representing an unexpected finding on this consensus. Such preference might be due to the experts’ perception that greater importance should be given to undesirable effects in evaluating the balance between benefits and harms compared with other clinical contexts involving PSMs that are different from PM-CRC.

The failure of OX-based HIPEC in PRODIGE 7 has many potential explanations other than the drug itself, and it should be emphasized that this trial only investigated the subset of CRC-PM patients who responded to neoadjuvant chemotherapy. Furthermore, the subgroup of patients with intermediate PCI (11–15), and also patients having upfront CRS/HIPEC, appear to benefit. Several groups have dissected the flaws of the high-dose OX-based HIPEC regimen in relation to the dose and duration of the perfusion.^43,54,64,65^ Preclinical data suggest higher cytotoxicity of longer duration despite using lower doses of OX-based regimens.^60,66^ Only one small (*n* = 29) clinical observational study compared OX 200 mg/m^2^ at 120 min of HIPEC with MMC-based HIPEC and found no difference. In R7, the experts preferred MMC 40 mg/m^2^ in this setting (77.5%, WP, LoE very low), but some caution is needed while interpreting the results of the consensus. At the time of the consensus, several other larger studies were ongoing, to investigate the longer duration of OX-based HIPEC regimens. The panel (Q14A, 90%) considers investigating these longer OX-based HIPEC regimens as a research pathway of importance.

The role of iterative CRS and HIPEC in PM-CRC, compared with palliative systemic treatment, is investigational. The panel indicated a role for iterative CRS+HIPEC in isolated recurrences, especially if the recurrence occurred >1 year after the index procedure (R9, WP, 95%, LoE low). Such recurrence is most likely a surgical failure at the time of the index procedure, not a failure of the HIPEC regimen. Tumor biology and the ability to reach a complete CRS during the iterative procedure should guide the decision making.^67^ The panel identified MMC as the drug of choice for iterative CRS+HIPEC (R10, WP, 93.8%, LoE low). In primary CRC, several risk factors for metachronous PM have been identified.^39,68^ As a result, ‘prophylactic’ HIPEC was previously investigated as a treatment strategy to prevent PM in this setting.^12,13^ Unfortunately, both trials used the same suboptimal OX-based HIPEC regimen as the PRODIGE 7 trial. Despite these shortcomings and negative trials, the panel still considers prophylactic HIPEC a vital research priority (Q11A, 65%). An RCT (Q11B, 81, 25%) with high-dose MMC is the preference for such research. One criticism against the Dutch trial^58^ was the employment of old systemic chemotherapy schedules in adjunction with CRS and HIPEC. Most CRC patients currently receive neoadjuvant FOLFOX systemic chemotherapy, raising concerns about the potential chemoresistance to OX for subsequent CRS+HIPEC. As such, the panel identified the lasting need (Q12A, 77.5%) for an RCT (Q12B, 88.75%) with MMC or an MMC+CDDP+HIPEC regimen (Q12C) in a CRS alone versus CRS+HIPEC trial design. The CAIRO-6 trial is currently investigating this protocol.^69^ The impressive progress of systemic chemotherapy in the past decade has been mainly due to multicycle, multidrug, personalized chemotherapy regimens. In this context, it should be no surprise that the panel considers investigating multidrug HIPEC regimens in an RCT or comparative study (Q15B, 98.75%) as an essential research line (Q15A, 75%).

Some major limitations of this expert consensus merit discussion. First, the available evidence for most questions was of low quality, indirect (extrapolated), or even inexistent. Therefore, an expert consensus is needed to provide guidance to clinicians for their daily practice. Second, the expert panel consisted mainly of surgical oncologists and providers of CRS and HIPEC. This bias explains, to a certain extent, the surprisingly high degree of consensus in favor of HIPEC, which is in clear contrast to National Comprehensive Cancer Network (NCCN) and European Society for Medical Oncology (ESMO) guidelines that do not recommend HIPEC. However, the expert panels of these respective guidelines were arguably also biased in the sense that very few (if any) PSM surgeons were involved in the process. Furthermore, it can be added that the involved PSOGI experts of this consensus consistently reported, by large, the best outcomes for CRC-PM patients, giving some legitimacy.

## Conclusion

The expert panel consensually agreed that HIPEC with regimens other than high-dose/short duration OX from PRODIGE 7 might still be a viable treatment strategy for CRC patients with completely resectable isolated PM. The high-dose MMC regimen was the most preferred regimen for current clinical use for treating primary and recurrent PM-CRC. Ongoing preclinical and clinical studies should help standardize HIPEC methodology and regimens in future.

### Supplementary Information

Below is the link to the electronic supplementary material.

Supplementary file1 (PDF 10 kb)

Supplementary file2 (PDF 11 kb)

Supplementary file3 (PDF 11 kb)
