# Supplementary material for: 2022 Peritoneal Surface Oncology Group International Consensus on HIPEC Regimens for Peritoneal Malignancies: Colorectal Cancer
Source: Ann Surg Oncol. 2023 Nov 8;31(1):567–76. doi: 10.1245/s10434-023-14368-5 (PMC10695877; doi:10.1245/s10434-023-14368-5)

Figure 5 (online appendix). HIPEC-regimen for repeat CRS and HIPEC for recurrent peritoneal metastases of colorectal cancer

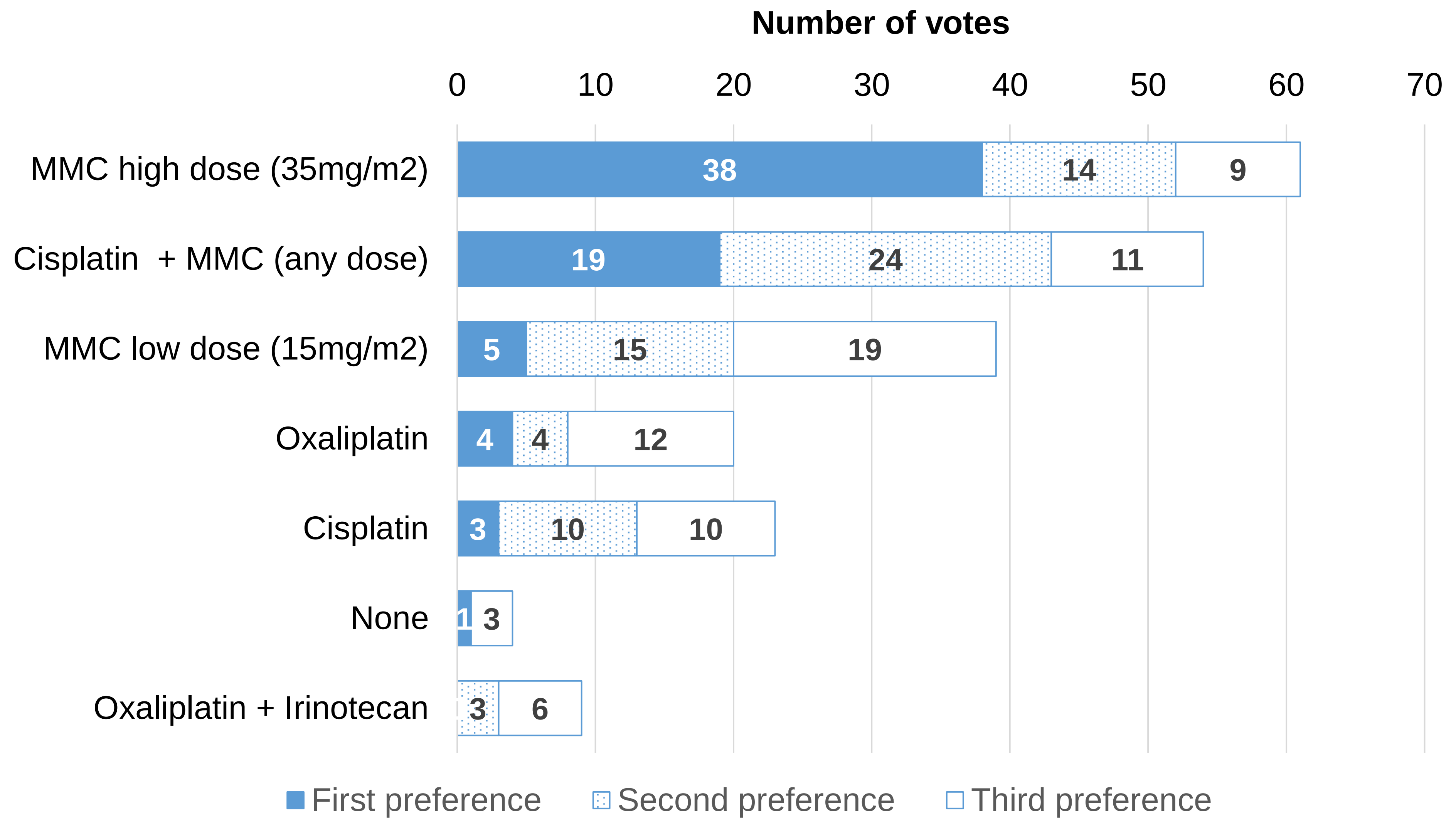

Supplement: Supplementary file 1 — Supplementary file1 (PDF 10 kb) [file 10434_2023_14368_MOESM1_ESM.pdf]
