# Supplementary figures and images for: 2022 Peritoneal Surface Oncology Group International Consensus on HIPEC Regimens for Peritoneal Malignancies: Colorectal Cancer
Source: Ann Surg Oncol. 2023 Nov 8;31(1):567–76. doi: 10.1245/s10434-023-14368-5 (PMC10695877; doi:10.1245/s10434-023-14368-5)

Figure 6. Priority HIPEC regimens for research proposal.

A. Treatment

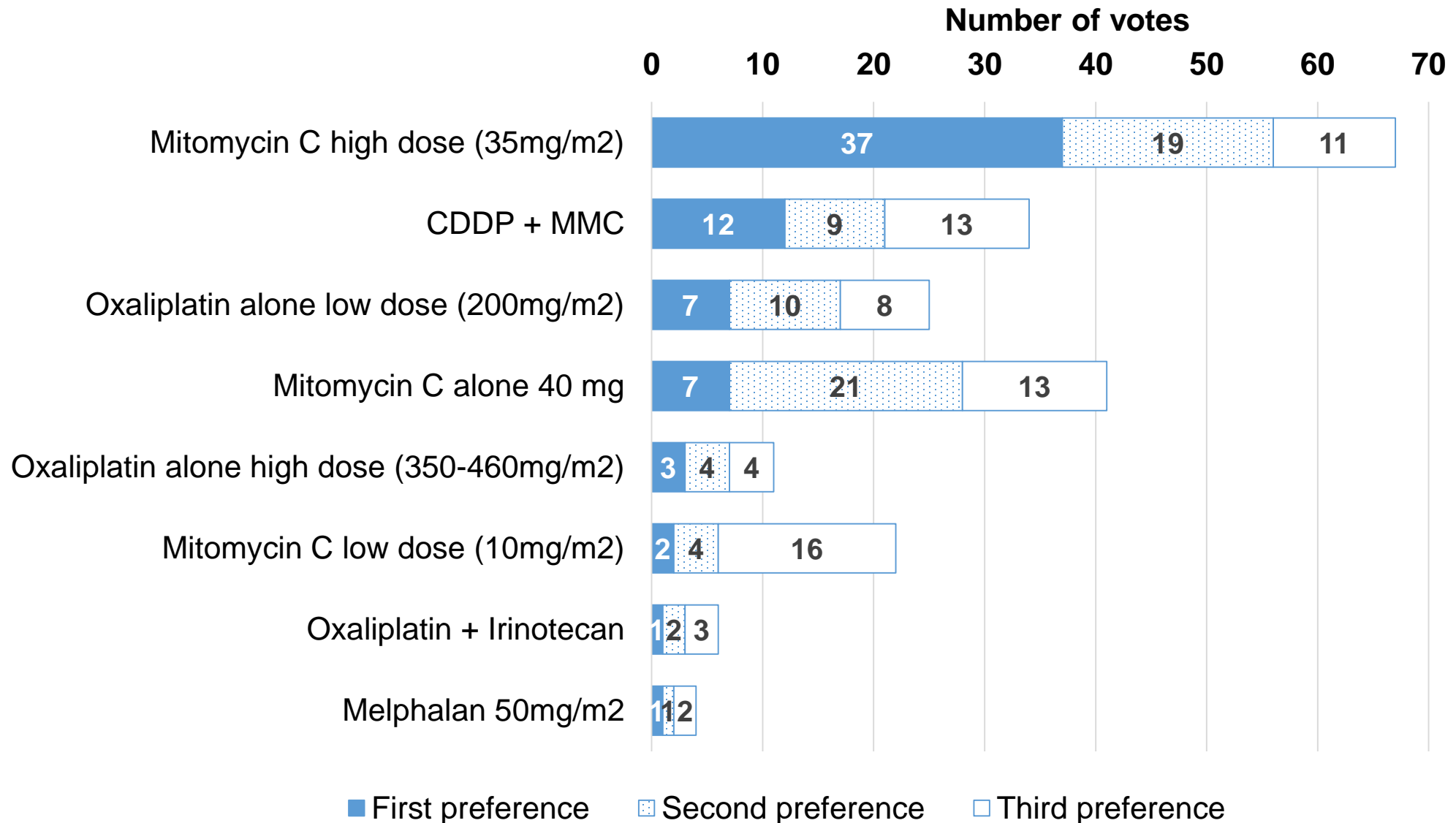

Supplement: Supplementary file 2 — Supplementary file2 (PDF 11 kb) [file 10434_2023_14368_MOESM2_ESM.pdf]
