# Supplementary material for: 2022 Peritoneal Surface Oncology Group International Consensus on HIPEC Regimens for Peritoneal Malignancies: Colorectal Cancer
Source: Ann Surg Oncol. 2023 Nov 8;31(1):567–76. doi: 10.1245/s10434-023-14368-5 (PMC10695877; doi:10.1245/s10434-023-14368-5)
Supplement: Supplementary file 3 — Supplementary file3 (PDF 11 kb) [file 10434_2023_14368_MOESM3_ESM.pdf]

B. Prophylaxis

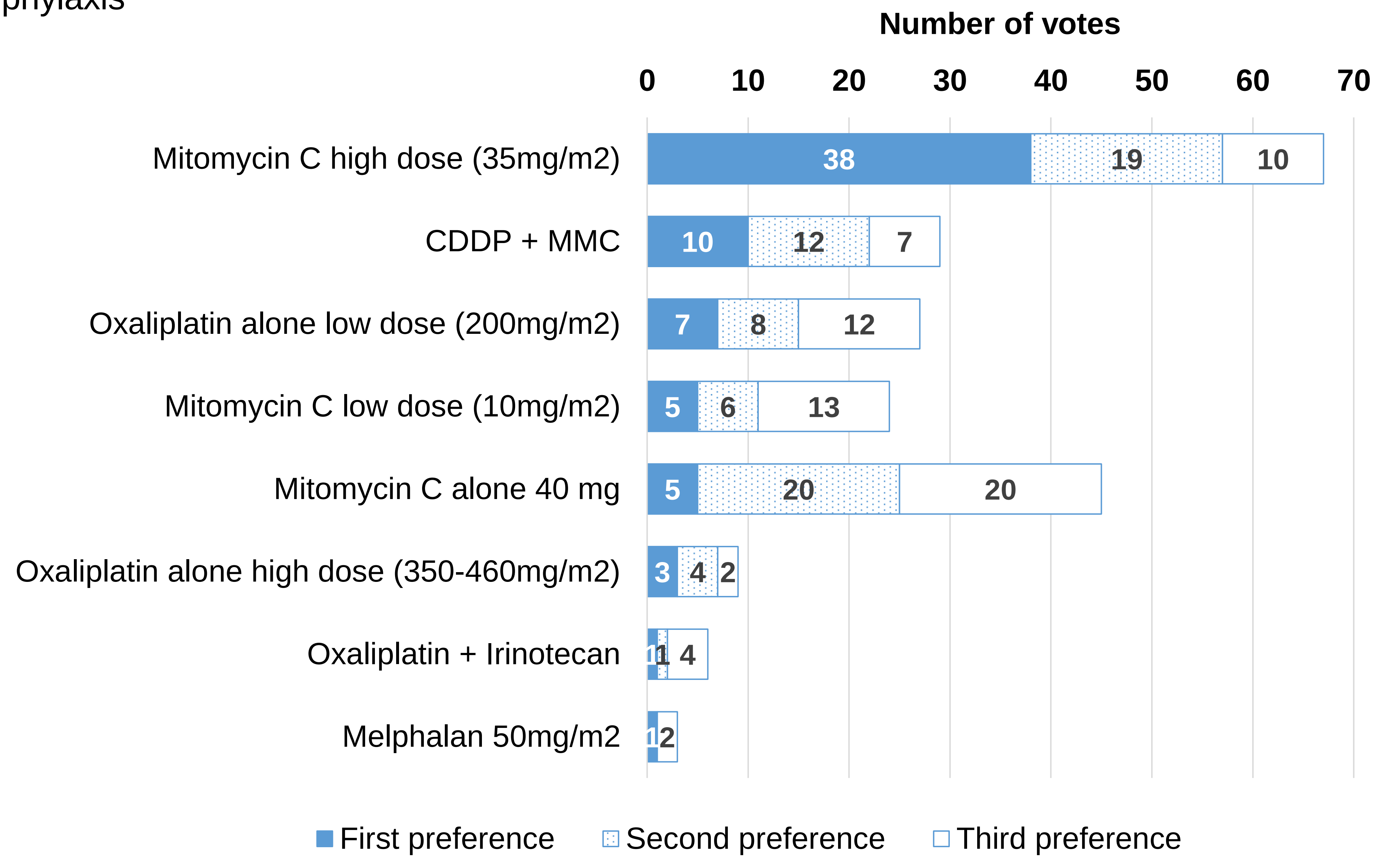

Panelists were provided a drop-down menu of eight HIPEC regimens and selected their preferred order of choice for (A) adjunct treatment or (B) prophylactic use. Depicted above are the number of 1st, 2nd and 3rd choice ratings for each regimen.
